# Clinical features, risk factors, and a nomogram for predicting refractory cervicogenic headache: a retrospective multivariate analysis

**DOI:** 10.3389/fneur.2025.1531180

**Published:** 2025-03-24

**Authors:** Jiawei Li, Baishan Wu, Xiaochen Wang, Lijuan Zhao, Jie Cui, Jing Liu, Kaikai Guo, Xiaoyu Zhang, Juan Liu

**Affiliations:** ^1^Department of Surgery, Beijing Huasheng Rehabilitation Hospital, Beijing, China; ^2^Department of Pain Medicine, Beijing Chaoyang Hospital Capital Medical University, Beijing, China; ^3^School of Medicine, Nankai University, Tianjin, China; ^4^Department of Pain Medicine, The First Medical Center of PLA General Hospital, Beijing, China; ^5^Department of Anesthesiology, Chengdu BOE Hospital, Chengdu, China

**Keywords:** cervicogenic headache, refractory headache, nomogram, risk stratification, multivariate analysis, clinical predictors

## Abstract

**Introduction:**

Given the intricate nature and varied symptoms of cervicogenic headache, its treatment can be challenging, potentially leading to refractory cervicogenic headache. We aimed to identify risk factors that could help predict the development of refractory cervicogenic headache in patients with cervicogenic headache.

**Methods:**

This is a retrospective cohort study of patients diagnosed with cervicogenic headache between January 1, 2022 and March 1, 2024 who underwent greater occipital nerve block. Data were collected by reviewing patients’ medical records and pain questionnaires. Covariates were selected using univariate and multivariate logistic regression analyses. A predictive nomogram model was developed to predict the unresponsiveness of the greater occipital nerves to anesthetic blockade.

**Results:**

Of the 82 patients studied, 46 experienced relief from headache following greater occipital nerve blocks, whereas 36 did not. In a multivariate analysis of patients with refractory cervicogenic headache, factors such as C2–C3 sensory loss [odds ratio (OR) = 13.10, 95% confidence interval (CI): 1.45–118.54], bilateral headache (OR = 7.99, 95% CI: 1.36–47.07), having two or more types of pain sources (OR = 5.51, 95% CI: 1.01–30.16), and limited cervical range of motion (>1) (OR = 13.05, 95% CI: 2.28–74.59) were identified as major prognostic indicators of unresponsiveness to greater occipital nerve blocks in cases of large occipital and cervical spine-related factors.

**Conclusion:**

Patients with severely limited cervical spine mobility, bilateral headaches, and C2–C3 sensory loss may not respond well to greater and lesser occipital nerve block therapy. Pain originating from multiple sources is typically associated with less favorable outcomes.

## 1 Introduction

Cervicogenic headache (CEH) is a type of headache originating from cervical spine disorders. These disorders involve various components, including bony structures, intervertebral discs, and soft tissues. CEH was first identified nearly 100 years ago and is typically characterized by a unilateral presentation, with pain usually felt on one side of the head without shifting to another. However, some patients may experience bilateral headache symptoms, indicating that the condition can manifest differently in different individuals (1, 2). In terms of prevalence, the occurrence of CEH within the general population ranges from approximately 0.17% to as high as 4.1% (3, 4). This figure indicates that while CEH is not the most common type of headache, it affects a notable portion of the population and warrants attention in clinical practice. From a semiological perspective, the characteristics of this persistent headache, which is of moderate to severe intensity and localized in the occipital, frontal, temporal, or orbital regions, exhibit a chronic pattern. This headache is often triggered by certain cervical postures. Owing to these overlapping features, they resemble both tension-type headaches and migraines. This similarity can complicate the clinical differentiation of CEH from other headache types, presenting challenges for accurate diagnosis and treatment in medical practice (4). Physiologically, CEH is a type of referred pain from the cervical spine that is similar to pain originating from the spine but felt in areas such as the shoulders, chest wall, buttocks, and lower extremities. The mechanism underlying CEH can be explained by trigeminocervical convergence, which involves the interaction between the cervical and trigeminal nerve fibers (1). This convergence enables pain signals from the upper cervical spine to be relayed to the head regions innervated by the cervical nerves (such as the occipital and auricular areas) and subsequently to the parietal, frontal, and orbital areas (5, 6). Previous studies have shown that structures innervated by the C1, C2, and C3 nerves can generate referred pain in the head (7). Additionally, even lower cervical spine pathologies below the C4 level can manifest as headaches (8, 9). This unique characteristic of CEH has attracted the interest of professionals from various disciplines beyond neurology, particularly pain specialists.

The diagnosis of CEH remains controversial because of the presence of clinical features associated with other headaches (10). Neurologists typically rely on clinical features for diagnosis. Currently, the standards for CEH mainly include the criteria established by the Cervicogenic Headache International Study Group (CHISG) in 1998 and the International Headache Society in 2018. The CHISG standard (11) is often used. Current treatments for CEH include medications (tricyclic antidepressants, gabapentin, or opioids), physical therapy (physiotherapy and exercise), acupuncture, interventional surgery (nerve tissue and radiofrequency therapy), and surgery (12–15).

The occipital nerve consists of the greater occipital nerve (GON) (derived from the posterior rami of C2 and C3) and lesser occipital nerve (derived from the anterior rami of C2 and C3), which are distributed in the skin of the occipital area, behind the auricle, and the lateral occipital area. The involvement of these nerves can result in regional pain and numbness (16, 17). The third occipital nerve originates from the posterior branch of the C3 nerve root and innervates the midline skin of the head and occipital region (18). Issues with the C2-C3 facet joint are the primary causes of CEH, accounting for approximately 70% of all cases (19–21). However, owing to lifestyle changes, CEH occurs at a younger age, and its symptoms become increasingly complex. Ultrasound-guided nerve block therapy often offers only short-term pain relief, leading to a long-term and recurring disease. In this study, we retrospectively gathered data from patients who underwent ultrasound-guided occipital nerve block, analyzed risk factors for poor efficacy of this nerve block, and aimed to offer insights into the selection of appropriate solutions for patients with CEH with different characteristics in clinical practice.

## 2 Materials and methods

In this retrospective study, we analyzed the medical data of patients treated at the Orthopedic and Joint Rehabilitation Department of Huasheng Rehabilitation Hospital between January 1, 2022 and March 1, 20,124. The inclusion criteria were diagnosis of CEH based on the 2018 International Classification of Headache Disorders, 3rd edition (2, 12) (Supplementary Table S1), and patients who were at least 18 years of age. Patients with incomplete data or those who were lost to follow-up were excluded. This study was performed in accordance with the guidelines of the Declaration of Helsinki and was approved by the Institutional Review Board of the Ethics Committee of the Beijing Huasheng Rehabilitation Hospital (approval no. 22–001). Written informed consent was obtained from all participants before treatment commenced. This process ensured that the participants were fully aware of the nature of the study and their rights, thereby adhering to the ethical standards required for research involving human participants. This study is compliant with STROBE Guidelines.

Quantitative data, including medical history, age, sex, duration of chronic exertional headache, and pain intensity levels before ultrasound-guided ablation surgery, immediately after surgery, and 1 month post-surgery, were collected from the registered department. Additionally, information on pain location and nature, negative life events, surgical history, trauma, and infection was collected. Questionnaires including the Self-Rating Depression Scale (SDS) and the 36-item Short-Form Health Survey (SF-36) were also administered. Pain intensity was assessed using the visual analog scale (VAS) (17).

If the pain intensity (VAS score) decreased by <30% and the quality of life score (SF-36) increased by <10% compared with results at the time of admission, the ultrasound-guided GON block was considered ineffective. Refractory cervicogenic headache (RCEH) is characterized by a lack of response to an ultrasound-guided GON block.

**Figure 1:**
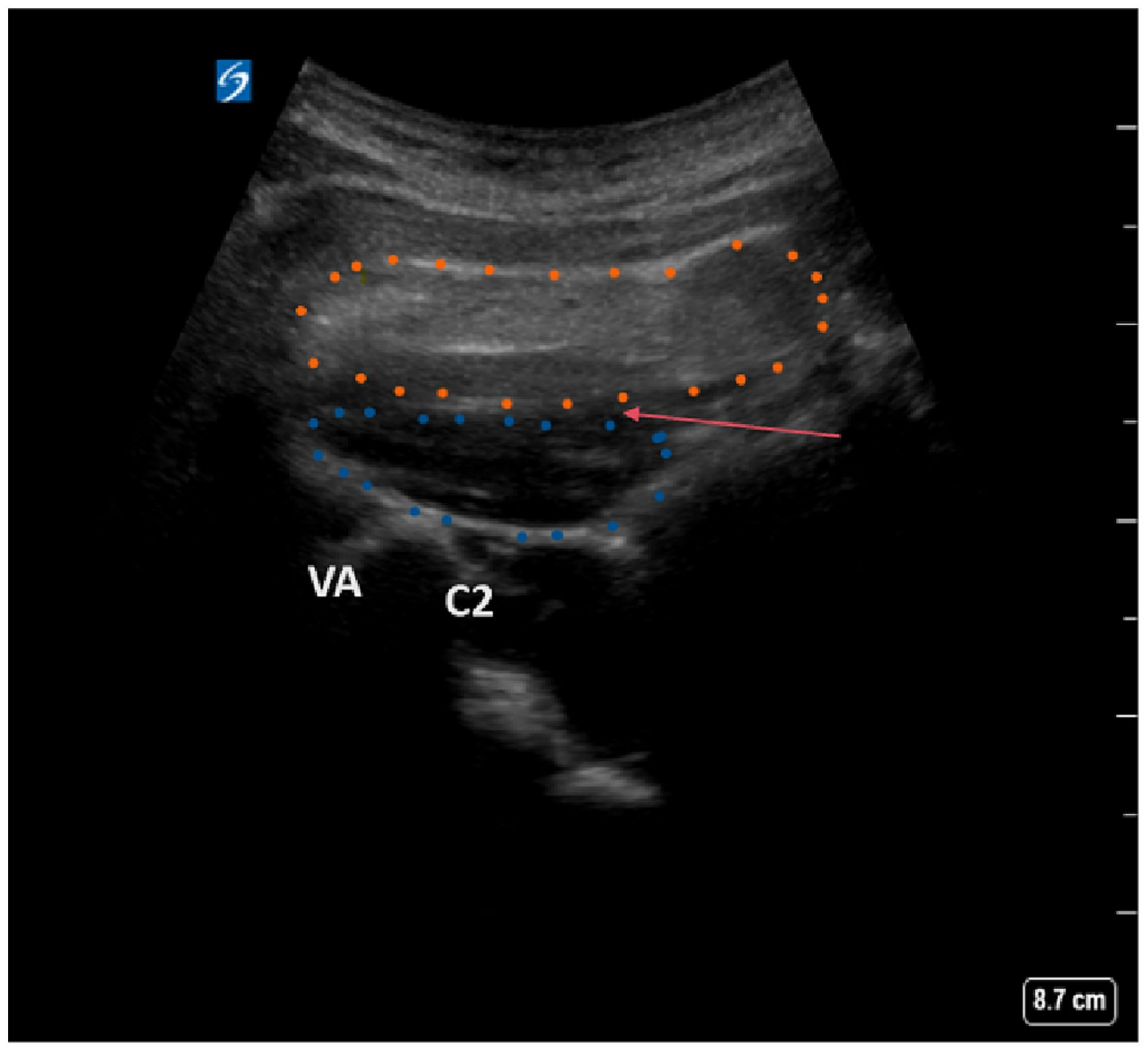
Ultrasound scanning plane and image of oblique capitis inferior. Blue dotted contour: oblique capitis inferior. Yellow dotted contour, musculi semispinalis capitis; C2, C2 spinous process; VA, vertebral artery; Red arrow, the puncture site.

A total of 82 patients underwent the ultrasound-guided GON block as part of the clinical procedure. Patients were positioned on their side, ensuring that the affected side was facing upward to facilitate access and visualization. The use of ultrasound imaging was critical in this procedure, enabling the precise identification of anatomical landmarks, including the C2 spinous process, vertebral artery, and inferior oblique, along with the semispinalis capitis muscles (Figure 1). The nerve block was strategically administered between the oblique capitis inferior and semispinalis capitis muscles, specifically targeting the GON based on its established anatomical positioning. To achieve local anesthesia, we administered a subcutaneous injection of 1% lidocaine hydrochloride. Subsequently, a 20G puncture needle, which was carefully guided by ultrasound imagery, was used to inject 10 mL of 0.2% lidocaine and 1 mL of 40 mg triamcinolone acetonide (Kenalog, Bristol-Myers Squibb, USA). The entire procedure was conducted under the guidance of ultrasound technology, specifically utilizing the Sonimage HS-1 ultrasound system (Konica Minolta Japan), outfitted with a 5-MHz curved probe designated L5-3. This meticulous approach was performed by Wu and Li, who possess substantial expertise in performing ultrasound-guided interventions, ensuring a high level of precision and safety throughout the procedure.

### 2.1 Statistical analysis

Data that followed a normal distribution are presented as mean ± standard deviation (SD), whereas non-normally distributed data are presented as median and interquartile range. Categorical data are presented as count and proportion. Logistic regression analyses were conducted to identify the independent variables associated with RCEH. Variables with *p* < 0.05 in the initial univariate analysis were incorporated in the multivariable model through the backward selection process. A significance level of *p* < 0.05 was utilized in the multivariate analysis. Statistical analyses were performed using the R software v3.6.0 (R Foundation for Statistical Computing, Vienna, Austria) with the random Forest SRC, party, party kit, and VIM packages.

## 3 Results

A total of 82 patients diagnosed with CEH were included in this study. The median age was 49.8 (range: 18–85) years, and most patients (53; 63.4%) were male. The median duration of pain experienced by the patients was 6.87 (range: 0.08–40) years. Additionally, 62.2% of patients had attained a bachelor’s degree or higher. On admission, the median intensity of pain as perceived by the patients was measured using the VAS and recorded at 7.09 ± 0.94 (mean ± SD). This intensity decreased to 4.08 ± 1.48 (mean ± SD) 1 month after discharge. Cervical spine involvement was identified as the source of headache in 25.6% of patients in the C1-2 segment, 90.2% in the C2-3 segment, and 24.4% in the C3-4 segment. Additionally, 21% of patients reported pain originating from two or more sources. Table 1 provides a descriptive analysis of the patients’ clinical characteristics. Major concomitant symptoms included radiating pain to the arm or shoulder in 48.8%, nausea or vomiting in 14.6%, dizziness in 25.6%, and memory deterioration in 4% of patients. Furthermore, 19 patients (23.2%) exhibited moderate-to-severe limitations in cervical range of motion (ROM >2). Sensory deficits in the C2–C3 region were found in 9 patients, representing 10.9% of the cohort. An aching pain was reported by 60 patients (73.2%) and radiating pain was experienced by 65 patients (79.3%). Insomnia was confirmed in 21 patients (25.6%). Of the 82 patients treated, 46 responded well to the ultrasound-guided GON block, as assessed using VAS scores and the SF-36 questionnaire, showing a significant response to the treatment. Conversely, 36 patients were identified as non-responders.

**Table 1:** Clinical and demographic characteristics.

Variable	All patients (*n* = 82)
Age (y), mean ± SD	49.79 ± 16.35
Sex, women, no (%)	53 (63.4)
Degree of education, no (%)	
Primary school degree	14 (17.1)
Secondary school degree	17 (20.7)
University diploma or higher	51 (62.2)
Characteristic of pain	
Aching pain	60 (73.2)
Dull pain	31 (37.8)
Radiating pain	65 (79.3)
Numbness	15 (18.3)
Throbbing pain	33 (40.2)
Region of pain, no (%)	
Cervical region	17 (20.7)
Occipital	70 (85.4)
Orbit	29 (35.4)
Ear	21 (25.6)
Parietal region	64 (78.0)
Frontal region	50 (60.9)
Pain at >3 regions	27 (32.9)
Source of pain	
C1–C2	21 (25.6)
C2–C3	74 (90.2)
C3–C4	20 (24.4)
Pain from >2 sources	29 (35.4)
Concomitant symptoms, no (%)	
Radiation of pain to arm or shoulder	40 (48.8)
Nausea or vomiting	12 (14.6)
Dizziness	21 (25.6)
Deterioration of the memory	3 (4)
More than two concomitant symptoms	23 (28.0)
Sensory deficit of C2–C3, no (%)	9 (10.9)
Limitation of cervical range of motion (ROM>2)	19 (23.2)
Duration of pain (≥1 years), no (%)	70 (85.4)
Bilateral headaches	20 (24.4)
Pain disrupts sleep	21 (25.6)
SDS, no (%)	
<53	12 (14.6)
53–62	26 (31.7)
63–72	36 (43.9)
>72	8 (10.0)

CEH, pudendal neuralgia; IQR, interquartile range; SDS, self-rating depression scale; ROM, range of motion.

**Figure 2:**
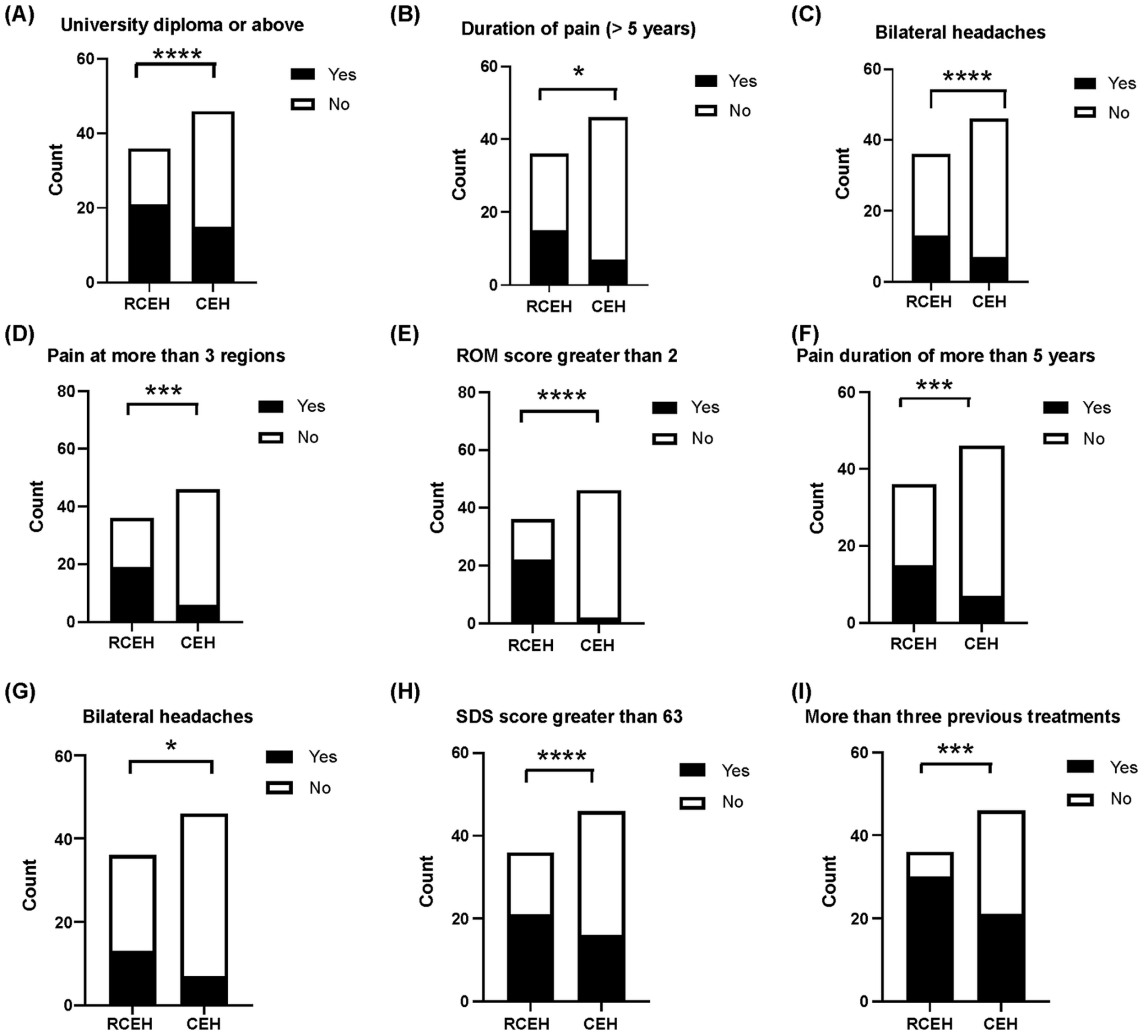
Univariate analyses regarding nonresponse to ultrasound-guided blocks of greater occipital and third occipital nerves. **(A)** Comparison of the number of patients with university education or above between those who did not respond and those who responded positively to the blocking of the greater occipital and third occipital nerves. **(B)** The number of patients with pain from more than one source. **(C)** Patients with two or more concurrent symptoms. **(D)**. Number of patients with pain at more than three regions. **(E)** Number of patients with limitation of cervical range of motion. **(F)** Number of patients suffering from CEH for more than 5 years. **(G)** Number of patients with bilateral headache. **(H)** Evaluation of patients with Self-Rating Depression Scale (SDS) scores exceeding 63 compared those who did not respond to those who responded positively to greater occipital and third occipital nerves block. **(I)** Assessment of patients who underwent more than three previous treatments. CEH, cervicogenic headache.

The results of the univariate analysis are depicted in Figure 2 and Table 2. From this analysis, the following nine potential risk factors were identified: having a university diploma or higher education, experiencing pain for more than 5 years, pain affecting more than three regions of the body, pain originating from multiple sources, more than concomitant symptoms, restricted cervical ROM (ROM >2), bilateral headaches, number of previous treatments, and an SDS score of >63. However, no significant differences were observed between the groups in terms of age, sex, pain radiating to the arm or shoulder, or insomnia.

**Table 2:** Univariate analysis of nonresponse to ultrasound-guided blocks of greater occipital and third occipital nerves.

Variable	OR (95% CI)	*p* value
Age (>65 vs. ≤ 65 years)	0.99 (0.37–2.58)	>0.99
Sex (women vs. men)	0.92 (0.39–2.17)	>0.99
Primary school degree	0.61 (0.24–1.80)	0.44
Secondary school degree	0.63 (0.29–1.79)	0.63
University diploma or above	11.56 (3.48–33.50) ^****^	<0.0001
Pain from >1 source	7.80 (2.82–20.24) ^****^	<0.0001
Radiation of pain to arm or shoulder	1.59 (0.65–3.65)	0.37
More than two concomitant symptoms	4.75 (1.79–13.59) ^**^	0.0021
Pain at >3 regions	9.39 (2.67–26.67) ^****^	<0.0001
Sensory deficit of C2–C3	2.87 (0.76–20.97)	0.17
Limitation of cervical range of motion (ROM >1)	1.22 (0.49–3.04)	0.8241
Limitation of cervical range of motion (ROM >2)	34.57 (7.60–155.3) ^****^	<0.0001
Duration of pain (>4 years)	2.27 (0.86–5.67)	0.1062
Duration of pain (>5 years)	5.01 (1.88–11.92) ^***^	0.0008
Bilateral headaches	3.15 (1.06–8.24) ^*^	0.04
Pain disrupts sleep	1.58 (0.59–4.05)	0.45
SDS		
>53	1.95 (0.63–5.51)	0.28
>63	2.63 (1.05–6.32) ^****^	<0.0001
Number of previous treatments		
>1	2.22 (0.63–6.88)	0.2478
>2	2.71 (0.84–7.41)	0.1135
>3	5.95 (2.17–17.26) ^***^	0.0006

^*^p < 0.05, ^**^p < 0.01, ^***^p < 0.01, ^****^p < 0.0001.RCEH vs. CEH group.SDS, self-rating depression scale; ROM, range of motion.

**Figure 3:**
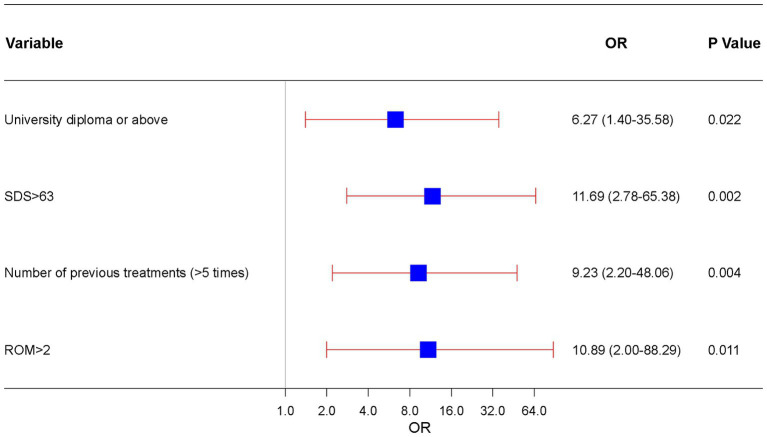
Independent risk forest plot for estimating nonresponse to ultrasound-guided greater occipital and third occipital nerve block.

**Figure 4:**
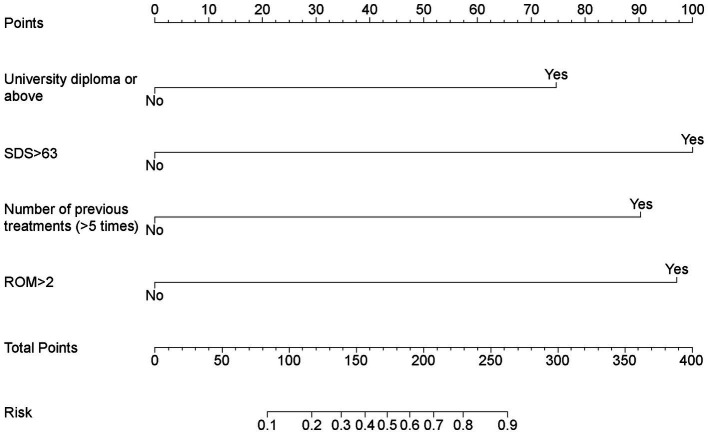
Nomogram for estimating nonresponse to ultrasound-guided greater occipital and third occipital nerve block.

Factors with *p* < 0.05 in the univariate analysis were included in the subsequent multivariate analysis. The results of the multivariate logistic regression analysis for RCEH are presented in Figure 3. Various factors were identified as potential predictors of the poor efficacy of ultrasound-guided greater and third occipital nerve blocks for CEH treatment. These factors include university diploma or above [odds ratio (OR): 6.27, 95% confidence interval (CI): 1.40–35.58], SDS > 63 (OR: 11.69, 95% CI: 2.78–65.38), number of previous treatments (>5 times) (OR: 9.23, 95% CI: 2.20–48.06), and ROM >2 (OR: 10.89, 95% CI: 2.00–88.29). The nomogram detailed in Figure 4 is intended to serve as a practical tool for clinicians to estimate the likelihood of nonresponse in patients undergoing this specific nerve block therapy.

## 4 Discussion

CEH is a common type of headache triggered by issues in the cervical spine, such as bony structures, disks, and soft tissues (2, 12), poses therapeutic challenges due to its heterogeneous etiology. The greater occipital nerve (GON), arising from the C2 spinal nerve, is a critical pain generator in CEH as it innervates the occipital region and converges with trigeminal afferents, facilitating pain referral to frontal areas (16). Ultrasound-guided GON blocks have become a first-line intervention, achieving short-term pain relief in 60–80% of cases by interrupting this nociceptive pathway (2, 15). In this study, we defined refractory CEH (RCEH) as persistent headache (VAS reduction <30%, score of SF-36 rise <10% 1 months post-surgery) following ultrasound-guided GON/third occipital nerve blocks, a criterion reflecting both the anatomical centrality of GON in CEH and clinical recognition of its therapeutic limitations. Among 82 CEH patients, 43.9% (36/82) developed RCEH, underscoring the need to identify predictors of nerve block resistance. We studied 82 CEH patients’ demographics and symptoms, assessing ultrasound-guided GON block effects on pain (VAS), depression (SDS), and quality of life (SF-36). Patients unresponsive to blocks were labeled RCEH, with 32 treated patients experiencing this. Univariate analysis found risk factors like education, pain sources, duration, headache side, depression, and past treatments. Multifactor analysis highlighted education, depression, prior treatment, and limited cervical movement as significant RCEH risk factors. A nomogram was developed to identify high-risk patients for advanced therapies.

Our findings align with prior studies emphasizing cervical spine dysfunction as a critical contributor to CEH refractoriness. For instance, 48.8% of the patients in our study experienced pain radiating to their arms and shoulders, which is consistent with the findings of prior studies (2, 7). The C2-3 joint is innervated by the GON, with cervical spine dysfunction causing 70% of CEH cases (22, 23); however, 90.2% of patients reported pain from the C2-C3 region, possibly influenced by sample size limitations. Additionally, sensory deficits in the C2–C3 region and nonresponse for the GON block may indicate neuropathic or compressive pathology at the upper cervical spine, such as occipital-cervical junction instability or facet joint arthropathy. This aligns with imaging studies demonstrating C2–3 degeneration in refractory CEH patients (23). However, our study uniquely highlights bilateral headache as a prognostic marker, a feature less emphasized in earlier CEH studies but consistent with observations in chronic migraine cohorts where bilateral pain predicts poorer treatment outcomes (24).

Patients had undergone multiple treatments before receiving this specific treatment, indicating the potential development of a complex CEH. Common clinical signs of CEH include reduced cervical spine mobility and strength, with a limited ROM in the neck as a diagnostic criterion (2). Our findings suggest that severe limitations in cervical spine mobility (ROM >1) indicate the ineffectiveness of a simple GON block. Another retrospective study identified limited cervical spine motion (ROM), higher Neck Disability Index scores, and increased risk factors for the surgical treatment of CEH (25). However, unlike previous studies that emphasized demographic factors such as gender, this study suggests that the majority of patients in the sample were male (63.4%). Univariate analysis revealed no significant differences in gender among refractory CEH patients, indicating that gender may not play a primary role in predicting treatment response. Our study identified education level and psychological distress (SDS >63) as novel predictors, suggesting psychosocial dimensions may modulate treatment response. Spinal disease is a common issue among college students, with prolonged sitting increasing risk and limiting motion (26). Literature and management majors may experience more cervical lateral flexion, indicating higher education’s role in complex CEHs (27). The identification of multisource pain as a risk factor extends prior work by Bogduk (1), who proposed that CEH arising from overlapping cervical segments (e.g., C1-2 and C2-3) creates complex nociceptive pathways resistant to isolated nerve blocks (1, 4). Notably, our results contrast with earlier reports that prioritized psychosocial factors (e.g., depression) as primary predictors of refractoriness. While we observed elevated SDS scores (>63) in univariate analysis, this variable did not retain significance in the multivariate model, suggesting that structural and biomechanical factors may dominate over psychological comorbidities in CEH progression.

Current CEH management adopts a hierarchical approach encompassing conservative, interventional, and surgical modalities (7). Conservative therapies, including oral medications (NSAIDs, tricyclic antidepressants, muscle relaxants) and physical therapy (manual manipulation, cervical stabilization exercises), serve as first-line interventions to address nociceptive and biomechanical contributors (28–30). While physical therapy aims to correct cervical dysfunction, its efficacy remains inconsistent, with studies reporting conflicting outcomes for pain reduction and functional improvement (31–33). Interventional options are prioritized for refractory cases. Ultrasound-guided nerve blocks targeting the greater occipital nerve (GON) or cervical dorsal ramus achieve short-term pain relief in 70–80% of patients, though benefits typically wane within weeks to months (34, 35). Advanced modalities like pulsed radiofrequency (PRF) and radiofrequency ablation (RFA) modulate nociceptive signaling through distinct mechanisms: PRF utilizes pulsed electromagnetic fields (42–46 MHz) to modulate nociceptive signaling, while RFA generates 60–80°C thermal lesions for neuroablative effects. While PRF offers superior safety, RFA provides longer-lasting analgesia but necessitates stringent patient selection to avoid permanent nerve damage (36–38). Botulinum toxin injections may benefit CEH patients with comorbid cervical dystonia, though variable efficacy and high costs limit their utility (39, 40). Surgical interventions (e.g., cervical fusion, minimally invasive decompression) are reserved for patients with confirmed structural pathologies (e.g., C2–3 radiculopathy, atlantoaxial instability) refractory to ≥6 months of conservative care. Approximately 60% of surgical candidates achieve sustained pain relief, but risks such as adjacent segment degeneration and infection demand meticulous patient counseling (41, 42).

Current CEH management relies on a stepwise approach, prioritizing minimally invasive interventions before advancing to neuromodulation or surgery (7). Our study identifies four robust predictors of refractory cervicogenic headache (RCEH): C2–C3 sensory loss, bilateral headache, multisource pain origins, and limited cervical range of motion (ROM). These factors hold significant clinical utility for optimizing CEH management. Patients with C2–C3 sensory deficits or ROM limitation likely exhibit advanced neuropathic or biomechanical pathology (e.g., facet arthropathy), necessitating early escalation to radiofrequency ablation (RFA) targeting cervical medial branches or combined pulsed radiofrequency (PRF) and physical therapy, rather than repetitive nerve blocks. For those with bilateral headache and multisource pain, interventions should address widespread nociceptive convergence through dual-target therapies (e.g., bilateral nerve blocks, neuromodulation). A stratified approach is critical: low-risk patients (unilateral, single-source pain) may respond to conservative measures, while high-risk subgroups (≥1 predictor) benefit from aggressive multimodal regimens. Integrating these predictors into clinical workflows enables personalized care, reduces futile treatments, and improves long-term outcomes—a paradigm shift toward phenotype-specific CEH management that future guidelines should formalize (Table 3).

**Table 3:** Proposed risk-stratified algorithm.

Risk profile	Recommended interventions
Low-risk (unilateral, single-source pain, and normal ROM)	Conservative therapy → GON block if refractory
High-risk (≥1 predictor: C2–C3 sensory loss, bilateral pain, multisource pain, and ROM >1)	Early RFA/PRF + physical therapy ± imaging → surgical evaluation if structural pathology is identified

ROM, range of motion; GON, greater occipital nerve; RFA, radiofrequency ablation; PRF, pulsed radiofrequency.

While our multivariate analysis did not identify educational attainment or occupation as independent predictors of refractory cervicogenic headache (RCEH), clinical observations suggest that highly educated individuals and those in cognitively demanding professions (e.g., teachers, lawyers) may exhibit heightened susceptibility to RCEH progression. Discrepancies may arise from biomechanical stress due to prolonged forward-head posture in sedentary jobs, increasing cervical load and pain incidence, and psychosocial factors like chronic stress and pain catastrophizing in educated individuals, which amplify pain perception (43–46). Additionally, The lack of statistical significance in our study may reflect unmeasured confounding factors, such as disparities in healthcare access that offset risks, or insufficient granularity of variables, including undifferentiated occupational ergonomics and broad educational classifications. Educational and occupational factors may exert their influence indirectly through mediating variables, such as limited cervical mobility and depression, rather than having a direct impact (47, 48). Therefore, clinicians should prioritize early multimodal intervention for educated patients with cervicogenic headache (CEH) to prevent refractory progression. Evidence shows that combining therapy reduces recurrence (49). Additionally, combning cognitive behavioral therapy improves compliance (50). Ergonomic optimization with transcranial magnetic stimulation increases SF-36 scores (51). Screening involves ergonomic evaluations, and treatment should start within three months post-diagnosis to maximize neuroplasticity in CEH management (52).

Moreover, the identified predictors—C2–C3 sensory loss, bilateral headache, multisource pain, and cervical range of motion (ROM) limitation—offer critical insights into the pathophysiology of refractory cervicogenic headache (CEH). C2–C3 sensory deficits may arise from neuropathic injury secondary to nerve root compression or occipital-cervical junction instability, potentially driven by pro-inflammatory cytokines (e.g., TNF-α, IL-6) released during degenerative cervical processes (53, 54). Bilateral headache and multisource pain likely reflect central sensitization, where persistent nociceptive input from multiple cervical segments (e.g., C1–C3 facet joints, myofascial tissues) amplifies thalamocortical signaling, perpetuating pain despite peripheral nerve blocks (55). Additionally, limited cervical ROM may signify biomechanical overload or facet joint arthropathy, promoting sustained nociception via mechanotransduction pathways involving PIEZO ion channels (56–58). The findings of this study also suggest the necessity of validating biomarkers, such as serum cytokine profiles and CSF neuropeptides like CGRP, as well as advanced imaging indicators, including dynamic MRI for instability and diffusion tensor imaging for axonal integrity, to stratify CEH subtypes. For instance, elevated levels of IL-1β or CGRP can differentiate inflammatory CEH from neurogenic CEH, while EEG-based cortical excitability patterns can identify the risk of central sensitization (59). Despite the limitations of this study, which include its retrospective design and the lack of mechanistic biomarker data, it outlines viable pathways for future research. These pathways include translational studies linking clinical predictors with molecular characteristics, such as transcriptomic analysis of cervical disc and facet joint tissues; prospective trials evaluating early anti-inflammatory treatments, such as IL-6 inhibitors, or neuromodulation techniques, like transcranial magnetic stimulation, in high-risk CEH patients; and mechanistic imaging that correlates cervical range of motion with facet joint PET-CT signals or spinal glial activation. By connecting clinical observations with pathophysiological hypotheses, this study advances targeted therapeutic strategies that address the multifactorial nature of CEH to some extent.

This study has some limitations. First, it was a retrospective cohort study, implying that a prospective randomized controlled study would provide a higher level of evidence and more meaningful results. Second, the generalizability of our findings is restricted, as the study participants were sourced from a single center with a small cohort. Studies with larger cohorts and data from multiple centers would enhance the credibility of the results. Furthermore, the scope of the study was limited because certain potential risk factors, such as muscle elasticity, vertebral artery blood flow, and other imaging characteristics, were not considered.

In conclusion, severe limitation of cervical ROM correlated with a worse prognosis of neurological diseases. Patients with bilateral headaches and sensory deficits in the C2-C3 region were prone to nonresponse to greater and lesser occipital nerve block treatments. Pain involving more than one source was associated with a poor prognosis.

## Data Availability

The original contributions presented in the study are included in the article/Supplementary material, further inquiries can be directed to the corresponding authors.
