# Supplementary material for: Clinical features, risk factors, and a nomogram for predicting refractory cervicogenic headache: a retrospective multivariate analysis
Source: Front Neurol. 2025 Mar 24;16:1531180. doi: 10.3389/fneur.2025.1531180 (PMC11973074; doi:10.3389/fneur.2025.1531180)
Supplement: Supplementary file 1 [file Table_1.DOCX]

Supplementary Material

# Supplementary Table

**Table S1**. IHS diagnostic criteria (3rd edition, 2018)

| 1. Any headache fulfilling criterion C |
| --- |
| 1. Clinical and/or imaging evidence of a disorder or lesion within the cervical spine or soft tissues of the neck, known to be able to cause headache |
| 1. Evidence of causation demonstrated by at least two of the following： |
| 1. Headache has developed in temporal relation to the onset of the cervical disorder or appearance of the lesion |
| 1. Headache has significantly improved or resolved in parallel with improvement in or resolution of the cervical disorder or lesion |
| 1. Cervical range of motion is reduced and headache is made significantly worse by provocative maneuvers |
| 1. Headache is abolished following diagnostic blockade of a cervical structure or its nerve supply |
| 1. Not better accounted for by another ICHD-3 diagnosis |

ICHD-3: International Classification of Headache Disorder, 3rd edition
